# Editorial: Risk Factors for Alzheimer's Disease

**DOI:** 10.3389/fnagi.2020.00124

**Published:** 2020-05-07

**Authors:** Ines Moreno-Gonzalez, Rodrigo Morales, David Baglietto-Vargas, Raquel Sanchez-Varo

**Affiliations:** ^1^Departamento Biologia Celular, Genetica y Fisiologia, Facultad de Ciencias, Instituto de Investigacion Biomedica de Malaga-IBIMA, Universidad de Malaga, Málaga, Spain; ^2^Networking Research Center on Neurodegenerative Diseases (CIBERNED), Madrid, Spain; ^3^Department of Neurology, The University of Texas Health Science Center at Houston, Houston, TX, United States; ^4^Centro Integrativo de Biología y Química Aplicada (CIBQA), Universidad Bernardo O'Higgins, Santiago, Chile

**Keywords:** Alzheimer's disease, risk factors, modifiable, genetic variance, peripheral

Late-onset sporadic Alzheimer's disease (AD) is a multifactorial disease in which several risk factors contribute to the onset and disease progression. Risk factors for AD include aging, sex, lifestyle, comorbidities, and genetic factors. Considering the progressive aging of the population, the prevention or delay of cognitive dysfunction by targeting modifiable risk factors is becoming a therapeutic approach of growing interest. In this Research Topic, we have compiled a series of manuscripts that describe emerging risk factors and the use of those for early diagnosis. In this line, Edwards et al. clearly summarize the relationship between treatable comorbidities, lifestyle habits and AD development. In this review, they expose data dealing with the preventive effects of moderate physical activity, a balanced diet and treatment of sleep disturbances. The effect of smoking and alcohol consumption to promote dementia is also debated as well as comorbid medical conditions linked to this neurodegenerative disease such as cerebrovascular diseases or depression. In this regard, Amtul et al. analyzed the effect of cerebrovasculature alterations, such as ischemia, and its causative link with the development of AD. It is remarkable the epidemiological data on how stress, as a common cause of depression, has an important role on inducing cognitive impairment, positing stress in the eye of the storm. In the same line, Ouanes and Popp remark the relationship between high cortisol levels and increased risk of cognitive decline and dementia. In fact, elevated cortisol not only affects AD pathology but can also worsen sleep and cardiovascular diseases. Modulation of glucocorticoids and hypothalamic-pituitary-adrenal (HPA) axis functioning may be considered as a pharmacological target. Conversely, there is an increasing number of reports indicating that long-term administration of benzodiazepines and related hypnotic drugs to treat depression or insomnia may induce cognitive dysfunction as a side effect, especially in elderly population. Ettcheto et al. debate on the relevance of sleep disorders in cognition and compile epidemiological clinical data, proposing that benzodiazepines may act as contributing factors to prompt cognitive decline in aged AD patients rather than patients that may be at higher risk are usually recommended to take them.

Although genetic variants are considered the primary cause for familial forms of AD, recent evidences indicate that genetic variants are also a prominent risk factor for sporadic AD cases, in addition to the apolipoprotein E (ApoE ε4 allele). Fernandez et al., Pietzuch et al., and Zhang et al. highlight several causative mechanisms by which APOE4 mediates both amyloid beta (Aβ) and tau pathology, altering brain connectivity and modulating the inflammatory process and glucose. Furthermore, these toxic effects triggered by ApoE are not only related to an isoform-specific manner (mainly the ε4 allele), but new evidences also suggest that multiple ApoE-related functions are specific to a particular cell type in the brain. In this regard, lipid dysregulation, deficient glucose metabolism and pro-inflammatory response in glia cells mediated by APOE are new exploratory means by which APOE may contribute to the development of AD and more deep understanding of these pathological processes could turn into new promising therapeutic targets for AD. Zhang et al. also emphasize other associated genes, such as the bridging integrator 1(BIN1) and the sortilin-related receptor 1 (SORL1), which modulate the amyloid and tau aggregates via alteration endosomal trafficking.

An interesting emerging model explaining the early events triggering AD involves microbial infection (Moir et al., 2018). Here, AD-associated Aβ would emerge after pathogen neuroinvasion. These misfolded protein aggregates known to have anti-microbial activity would act as a primary barrier of defense, helping to clear pathogens from the brain (Soscia et al., 2010). Complementary to this hypothesis, bacterial infection also leads to peripheral inflammation and blood-brain barrier (BBB) dysfunction. Interestingly, both events are known AD risk factors and could act in conjunction or separately to initiate the deleterious molecular pathways leading to AD. These facts are nicely discussed by Giridharan et al.. Moreover, the authors postulate that some outcomes derived from infection (e.g., sepsis) may posit risks for dementia. Several lines of investigation supporting this hypothesis are critically discussed in this article. Following the role of peripheral tissue alterations and AD, research on liver dysfunction as a potential risk factor for dementia has been importantly neglected. Most of the Aβ generated in the body is cleared by the liver (Hone et al., 2003) and eliminated by the urine (Ghiso et al., 1997). In that sense, it is surprising that little research has been done in this area. Estrada et al. summarize direct and indirect evidence supporting the role of liver damage specifically focused in non-alcoholic fatty liver disease and related BBB dysfunction in the progression of AD. Linked to the review by Giridharan et al. the authors also describe the role of certain pathogens in liver health and their possible involvement in AD by altering peripheral Aβ clearance.

At present, no pre-symptomatic diagnostic tests are available for AD. In that sense, several non-invasive approaches linking peripheral changes and dementia have been explored. Positive associations have the potential benefit to help in clinical trials enrolment and implementation of preventive treatments in the future. Nishizawa et al. describe that direct measurement of abdominal visceral fat is inversely related to cognitive decline. This result supports previous epidemiological data and has the advantage of being directly performed by weighting the actual adipose tissue at short post-mortem time windows. Similarly, Liu et al. communicate that grip strength is linked to cognitive function in a large Chinese cohort. Although attractive, it is clear that confounding factors other than motor tests or physical functions may be responsible for the observed outcomes.

In addition to the different risk factors that have been identified and that provide prospects to determine the potential to develop AD, novel clinical approaches and biomarkers are emerging in an effort to monitor the onset and progression of AD pathology and therefore, be used as early diagnostic assessments. In this line, Qi et al. propose the evaluation of the functional connectivity in the medial temporal lobe as a measurement of initial memory impairment related with AD, providing a novel imaging biomarker to predict and diagnose AD. On a similar line, Amtul et al. provide with new evidences about the usefulness of imaging microvascular alterations to determine the severity of AD pathology. In a very compiling study, Sapkota et al. present a variety of biomarkers for AD, including salivary biomarkers in conjunction with other risk markers, and combine them in a multi-modal array analysis to determine their collective ability to discern and even predict the clinical status of AD patients and mild cognitive impairment from cognitively normal individuals.

Overall, all these studies point out the necessity to identify potential risk factors of AD, emphasizing the interrelation of multiple of these factors and the utility of combining a variety of markers to better predict and early diagnose AD. Although modifiable risk may be somehow prevented or controlled, genetic variations combined with other potential factors may hinder AD prevention. Further understanding on the interrelationship of these biomarkers will facilitate personalized therapeutic interventions in individuals at risk.

## Author Contributions

IM-G, RM, DB-V and RS-V have contributed to manuscript writing and editing. IM-G coordinated, reviewed, and approved the final version. All authors have made a substantial intellectual contribution to this manuscript and approved it for publication.

## Conflict of Interest

The authors declare that the research was conducted in the absence of any commercial or financial relationships that could be construed as a potential conflict of interest.
